# Association of the single nucleotide polymorphism in CAPN3 gene with growth performance in Merino and Garut (MEGA) backcross sheep

**DOI:** 10.1186/s43141-023-00524-7

**Published:** 2023-07-17

**Authors:** Puruhita, Ronny Rachman Noor, Endang Tri Margawati, Herman W. Raadsma

**Affiliations:** 1 Faculty of Animal Science, IPB University, Bogor, 16680 Indonesia; 2 Department of Animal Production and Technology, Faculty of Animal Science, IPB University, Bogor, 16680 Indonesia; 3 Research Centre for Applied Zoology, National Research and Innovation Agency, BRIN, Jalan Raya Bogor KM. 46, Cibinong, Bogor, 16911 West Java Indonesia; 4 Center for Advanced Technologies for Animal Genetics and Reproduction, Faculty of Veterinary Science, University of Sydney, Camden, NSW 2006 Australia

**Keywords:** MEGA sheep, PCR–RFLP, CAPN3 gene, BseSI, SNP, Growth traits

## Abstract

**Background:**

Sheep is one of the commodities of livestock which has been known widely in Indonesia for supporting the national food security. Improvement in genetic quality by selection based on genetic markers for growth is necessary to increase meat production. Quantitative trait loci (QTL) analysis in sheep suggests that Calpain 3 gene (CAPN3) gene might be one of the candidate loci affecting growth traits. CAPN3 is located on chromosome 7 sheep expressed in the skeletal muscles. The aim of this study was to investigate polymorphism CAPN3 intron 11 in Merino × Garut (MEGA) backcross using the PCR-RLFP method and to determine their association with growth traits.

**Results:**

SNP intron 11 CAPN3 | BseSI of Merino × Garut (MEGA) backcross sheep was polymorphic and resulted in two alleles of C and T with a frequency of 0.76 and 0.24, respectively, and CC, CT, and TT genotypes with a frequency of 0.54, 0.43, and 0.02, respectively. These loci were found to be in Hardy–Weinberg equilibrium. The SNP CAPN3 | BseSI significantly affected (*P* < 0.05) the birth weight in Merino × Garut (MEGA) backcross sheep.

**Conclusion:**

This result suggests that the CAPN3 | BseSI can be used as a genetic marker for birth weight trait in sheep.

## Background

Sheep is one of the commodities of livestock which has been known widely in Indonesia. Sheep farming is one alternative to animal protein sources for supporting national food security. Improvement of genetic quality by selection based on genetic markers for growth is necessary to increase the farmer’s income. Improvement of genetic quality is determined by the strength of inheritance and genetic quality of the traits that are improved. Selection using genetic markers is commonly performed to improve productivity in the livestock industry.

The recent developments in molecular genetics have found a possible method to identify association genetic variation at specific loci affecting the quantitative trait. Quantitative trait loci (QTL) analysis in sheep suggests that Calpain 3 gene (CAPN3) gene might be one of the candidate loci affecting growth traits [1]. Growth traits in livestock are one of the important quantitative traits because it is associated with carcasses. CAPN3 located on chromosome 7 sheep is expressed in the muscles. CAPN3 (calcium-activated neutral protease), also known as p94, is mostly expressed in the skeletal muscle, where it plays an important role in the integrity of myofibril [2]. CAPN3 has an important role in regulating protein degradation and controlling muscle growth which reflects the combined activity of protein synthesis and protein degradation [3]. The SSCP pattern showed three allele sequences of CAPN3 in sheep, and single nucleotide polymorphism (SNP) was identified at exon 10 [1]. Furthermore, CAPN3 SNP on intron 11 was found to be associated with birth weight in sheep [4].

The sheep used in this study were Merino and Garut (MEGA) backcross sheep. Garut sheep is a local sheep from West Java, and it has a medium type. Merino sheep are large types of sheep, and it is originally from Spain. Crosses of two sheep strains that have different genetic traits are used to form heterozygous populations, so it could find the allelic variations. Backcross can predict that phenotypically distinct sheep lines also have different alleles [1]. Therefore, it is necessary to know the diversity of the CAPN3 gene genotype in MEGA backcross sheep and their associations with growth traits. This study aimed to study the polymorphism of the CAPN3 gene in Garut and Merino backcross sheep and its association with growth traits.

## Methods

### Sample collection and DNA extraction

The sample of 85 Merino and Garut (MEGA) backcross sheep was obtained from F1 sires (Garut × Merino) which then were crossed back to Merino (MM) [1]. The growth trait dataset includes body weight (BW) of birth weight (BW0), 90 (BW90), 180 (BW180), 270 (BW270), and 360 (BW360) days of age. DNA was extracted from sheep white blood cells of fresh blood samples which were taken from the jugular vein. DNA was extracted based on a modified method of Montgomery dan Sise [5].

### SNP marker genotyping

SNP polymorphisms of the sheep CAPN3 gene were genotyped by the polymerase chain reaction-restriction fragment length polymorphism (PCR–RFLP) method. A pair of specific PCR primers, forward primer 5′-GACGAGCTTCAGACCACCTC-3′ and reverse primer 5′-CTGGTAACGCTGCACACACT-3′, was designed to amplify a 380-bp fragment of the intron 11 of sheep CAPN3 gene. PCR primers were designed based on the CAPN3 gene sequences (CAPN 3 sheep genome sequence ENSOARG00000020529) using the Primer 3.0 software. PCR amplifications were performed in a 12.5-µL reaction containing 0.5 µL template DNA, 6.25 µL Kappa Taq PCR kit, 4.75 µL nuclease-free water, and 0.5 µL for each forward and reverse primers. The PCR reaction was optimized in the Techne TC-PLUS. The reaction was cycled with the following conditions, initial denaturation for 5 min at 94 °C followed by 35 cycles of denaturation at 94 °C for 1 min, annealing at 59.7 °C for 30 s, and extension at 72 °C for 30 s and final extension at 72 °C for 5 min. PCR products were visualized by electrophoresis on 1% agarose gel in 0.5 × TBE. The PCR products were digested with BseSI enzyme that identified the GTGCC|C restriction site for genotyping; 3 µl of PCR product was digested with 0.7 µl buffer g, 0.3 µl (10 u/µl), and 5.8 µl DDW up to a total volume of 10 µl then were incubated at 55 °C for 2 h. The size of the restriction fragments was determined by electrophoresis on 2% agarose gel in 0.5 × TBE and visualized by ethidium bromide staining for ± 50 min at 100 V.

### Statistical analysis

Allele frequencies, gene frequencies, observational heterozygosity (Ho), and expected heterozygosity (He) were calculated by the direct counting method [6], and Hardy–Weinberg equilibrium in a population was tested using a chi-square (*X*^2^) test. Pearson’s correlation coefficients were calculated to test the strength of the relationship between growth traits of birth weight, body weight at 90 days, body weight at 180 days, body weight at 270 days, and body weight at 360 days. The association of genotypes with the growth traits was analyzed using the *t*-test. Phenotype data in three genotypes with significant differences of *P* < 0.05 by the *t*-test were detected as significant SNP.

## Results

### Polymerase chain reaction amplification of intron 11 Gen CAPN3

**Fig. 1:**
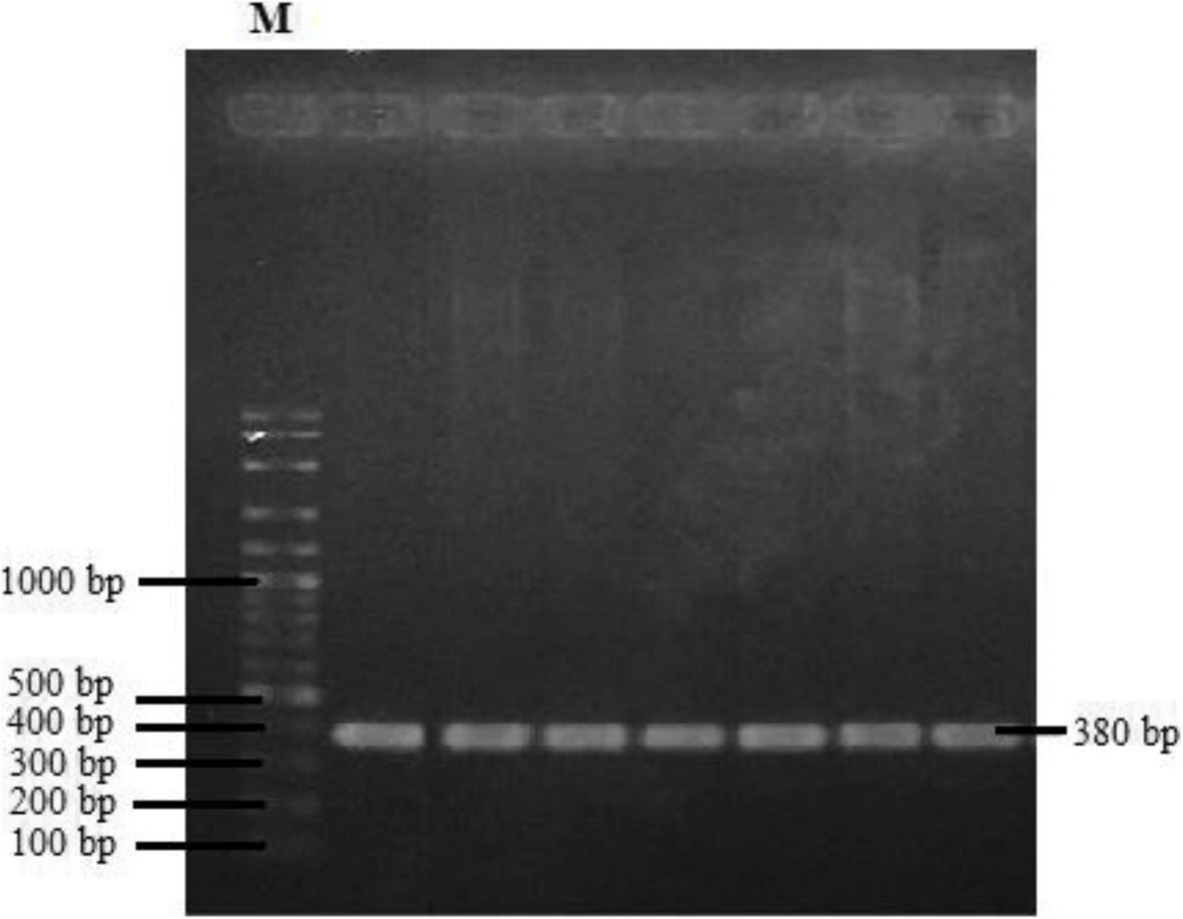
Visualization of PCR product of CAPN3 analyzed by electrophoresis. M, marker 100 bp

CAPN3 gene fragments were successfully amplified using MEGA backcross sheep’s DNA and pair of primers. The PCR products were detected by electrophoresis on 1% agarose gels with a 100-bp DNA marker (Fig. 1).

### Genotype identification and genetic diversity analysis

**Fig. 2:**
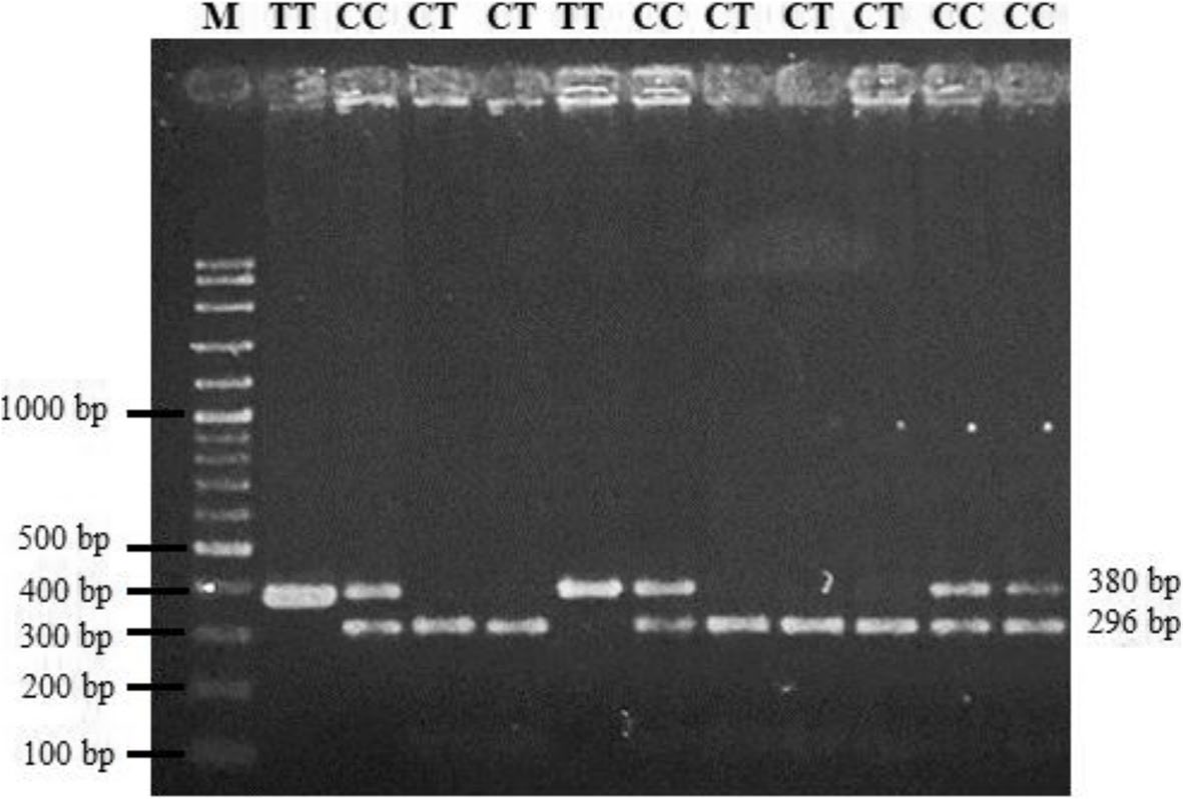
Results of PCR–RFLP of the SNP CAPN3|BseSI. M, marker 100 bp

SNP of sheep CAPN3 gene was genotyped by the polymerase chain reaction-restriction fragment length polymorphism (PCR–RFLP) method. Three different genotypes of the locus could be obtained by this method. The PCR product of 380 bp length was digested by restriction enzyme BseSI and produced fragments lengths of 296 bp and 84 bp for the CC genotype; 380 bp, 296 bp, and 84 bp for the CT genotype; and 380 bp for the TT genotype (Fig. 2). The 84-bp band is not visible on the gel because it is too small in size (< 100 bp). The genotype and allele frequencies of CAPN3|BseSI in MEGA backcross sheep are shown in Table 1.

**Table 1:** Genotype and allele frequencies and *X*^2^ test for CAPN3|BseSI locus

Genotype frequency			Allele frequency		Ho	He	*X*^2^	HW
CC (*n*)	CT (*n*)	TT (*n*)	C	T				
0.54 (46)	0.43 (37)	0.02 (2)	0.76	0.24	0.43	0.36	2.88	ns

*n* number of individuals, *ns* non-significant (*P* > 0.05)

### Association of SNP CAPN3|BseSI with growth traits

The results of association analysis between single nucleotide polymorphism (SNP) at intron 11 CAPN3 gene and growth trait of MEGA backcross sheep are shown in Table 2.

**Table 2:** Association of SNP CAPN3|BseSI and growth traits

Trait	Genotype means ± SE			*T*-test value		
	CC (*n* = 46)	CT (*n* = 37)	TT (*n* = 2)	CC vs CT	CC vs TT	CT vs TT
BW0	4.04 ± 0.73^a^	4.35 ± 0.72^b^	4.36 ± 0.09^b^	− 1.98^*^	− 0.63^ ns^	− 0.02^ ns^
BW90	14.15 ± 2.99^a^	13.41 ± 2.79^a^	16.66 ± 1.74^a^	1.15^ ns^	− 1.17^ ns^	− 2.47^ ns^
BW180	22.97 ± 5.10^a^	21.87 ± 4.95^a^	27.72 ± 3.41^a^	0.99^ ns^	− 1.30^ ns^	− 1.64^ ns^
BW270	29.29 ± 6.53^a^	27.78 ± 6.41^a^	33.03 ± 2.77^a^	1.06^ ns^	− 0.80^ ns^	− 1.14^ ns^
BW360	32.83 ± 7.52^a^	31.43 ± 7.27^a^	34.94 ± 1.90^a^	0.86^ ns^	− 0.39^ ns^	− 0.67^ ns^

*BW0* Birth weight, *BW90* Body weight at 90 days, *BW180* Body weight at 180 days, *BW270* Body weight at 270 days, *BW360* Body weight at 360 days

^a,b^Least square mean within a row with different superscripts differ significantly

### Correlations between various body weights

The correlations between body weight in this study were all positive (Table 3) which showed that the selection for weight would have a positive effect on weights at later ages.

**Table 3:** Pearson correlation coefficients between body weight at selected days of age

Trait	BW0	BW90	BW180	BW270	BW360
BW0	1				
BW90	0.259^*^	1			
BW180	0.254^*^	0.905^**^	1		
BW270	0.187	0.798^**^	0.912^**^	1	
BW360	0.212	0.751^**^	0.877^**^	0.945^**^	1

*BW0* Birth weight, *BW90* Body weight at 90 days, *BW180* Body weight at 180 days, *BW270* Body weight at 270 days, *BW360* Body weight at 360 days

^*^P < 0.05

^**^P < 0.01

## Discussion

The DNA amplification with an annealing temperature of 57.9 °C produced a single band according to the target size and not formed the band produced by non-specific amplicons (Fig. 1). An annealing temperature which is too high can produce a low PCR product, and an annealing temperature which is too low tend to stick elsewhere and produce non-specific products [7]. The amplified fragment size was consistent with the target which was 380 bp in length and had a good specificity (Fig. 1).

The genotype frequencies for CC, CT, and TT were 0.54, 0.43, and 0.02, respectively, and the frequency of the C allele was 0.76 and that of the T allele was 0.24 (Table 1). The frequency of the CAPN3 gene alleles was estimated as polymorphic. An allele was estimated as polymorphic if the allele frequency obtained is more than 0.01 [6, 8, 9]. The diversity of genes can be used as a reference in determining breeding programs that are selected when the population is diverse and crossing if the population is uniform [10]. The genetic diversity indicated a higher genetic variation and a selective potentiality which could be expected to gain more genetic progress [11]. The observed heterozygosity (Ho) and expected heterozygosity (He) values are 0.43 and 0.36, respectively (Table 1). The result of the chi-square test indicates that these loci were fitted in a Hardy–Weinberg equilibrium (*P* > 0.05).

The SNP on intron 11 CAPN3 gene had significantly affected (*P* < 0.05) the birth weight in MEGA backcross sheep (Table 2). Previous studies have shown that intron regions play an important role in the level of gene expression [5, 12–14]. Therefore, the specific nucleotide can be used as a genetic marker for birth weight selection. Animals with the CT genotype had higher birth weights than animals with the CC genotype. Previous studies have been reported that a CAPN3 polymorphism was associated with birth weight in sheep [5, 14]. Calpain activity is required for myoblast fusion and for cell proliferation, in addition to cell growth. The calpain system may also affect the number of skeletal muscle cells in domestic animals by altering the rate of myoblast proliferation and modulating myoblast fusion [15]. However, there was no significant effect (*P* > 0.05) of SNP CAPN3|BseSI genotypes on body weight at BW90, BW180, BW270, and BW360. As stated by Gardner et al. [16], birth weight is important for the next growth since it has a correlation in neonatal and adult health. It was reported that there is a benefit of ewe lambs mating; it could improve lifetime production, increase rates of genetic gain, and increase net profits [17]. In addition, it can only be achieved when the ewe lambs successfully rear their offspring for weaning. Another study indicated that genetic gain can be increased by selecting from ewe lambs (8-9 months) [18]. The birth weight was different between those produced by ewe lambs and mature ewes which the birth weight was heavier in mature ewes than in ewe lambs [19]. Another research reported that a low body weight would increase the deposition of fat in the carcass and non-carcass components during the period of lamb fattening [20].

The highest correlation was found between BW270 and BW360 (0.945) while the lowest correlation was found between BW0 and BB270 (0.187) (Table 3). The correlation coefficients between adjacent ages were high and decreased slightly as the interval between ages increased. These results are similar to the genetic correlation and correlation of phenotypic weight at different ages in sheep [21–23], cattle [24], and swine [25] which shows that the correlation coefficient between adjacent ages is high and decreases due to increasing age range. In this study, correlation coefficients between BW0 and other weights were lower than the corresponding values observed between weights at adjacent ages. This shows that birth weight is not under the same genetic control as weight at other ages [21]. Birth weight affects further growth traits [16]. Growth traits are quantitative traits and are controlled by multiple micro-effect genes [26]. In addition, it was stated that molecular markers related to sheep growth traits are then used as the basis of molecular breeding.

## Conclusion

SNP intron 11 of CAPN3|BseSI gene in MEGA backcross is polymorphic, and the C allele had higher frequencies compared to the T allele. The SNP intron 11 of CAPN3|BseSI gene in MEGA backcross sheep significantly affected (*P* < 0.05) for birth weight, and it could be used as a genetic marker for birth weight trait in sheep.

## Data Availability

All data are primary data and generated from the research.

## Abbreviations

QTL: Quantitative trait loci
CAPN3: Calpain 3
MEGA: Crossing Merino × Garut sheep
BW: Body weight
BW0: Birth weight
BW90: Body weight 90 days
BW180: Body weight 180 days
BW270: Body weight 270 days
BW360: Body weight 360 days
PCR: Polymerase chain reaction
PCR-RFLP: Polymerase chain reaction-restriction fragment length polymorphism
SNP: Single nucleotide polymorphism
